# WIDDE: a Web-Interfaced next generation database for genetic diversity exploration, with a first application in cattle

**DOI:** 10.1186/s12864-015-2181-1

**Published:** 2015-11-14

**Authors:** Guilhem Sempéré, Katayoun Moazami-Goudarzi, André Eggen, Denis Laloë, Mathieu Gautier, Laurence Flori

**Affiliations:** CIRAD, UMR INTERTRYP, F34398 Montpellier, France; INRA, UMR 1313 GABI, F78350 Jouy-en-Josas, France; Illumina Inc., Hayward, CA USA; INRA, UMR CBGP (INRA/CIRAD/IRD/Supagro), F34988 Montferrier-sur-Lez, France

**Keywords:** Polymorphism, SNP, Genetic diversity, Principal component analysis, NoSQL, Allele sharing distance, Population assignment, Cattle

## Abstract

**Background:**

The advent and democratization of next generation sequencing and genotyping technologies lead to a huge amount of data for the characterization of population genetic diversity in model and non model-species. However, efficient storage, management, cross-analyzing and exploration of such dense genotyping datasets remain challenging. This is particularly true for the bovine species where many SNP datasets have been generated in various cattle populations with different genotyping tools.

**Description:**

We developed WIDDE, a Web-Interfaced Next Generation Database that stands as a generic tool applicable to a wide range of species and marker types (http://widde.toulouse.inra.fr). As a first illustration, we hereby describe its first version dedicated to cattle biodiversity, which includes a large and evolving cattle genotyping dataset for over 750,000 SNPs available on 129 (89 public) different cattle populations representative of the world-wide bovine genetic diversity and on 7 outgroup bovid species. This version proposes an optional marker and individual filtering step, an export of genotyping data in different popular formats, and an exploration of genetic diversity through a principal component analysis. Users can also explore their own genotyping data together with data from WIDDE, assign their samples to WIDDE populations based on distance assignment method and supervised clustering, and estimate their ancestry composition relative to the populations represented in the database.

**Conclusion:**

The cattle version of WIDDE represents to our knowledge the first database dedicated to cattle biodiversity and SNP genotyping data that will be very useful for researchers interested in this field. As a generic tool applicable to a wide range of marker types, WIDDE is overall intended to the genetic diversity exploration of any species and will be extended to other species shortly. The structure makes it easy to include additional output formats and new tools dedicated to genetic diversity exploration.

**Electronic supplementary material:**

The online version of this article (doi:10.1186/s12864-015-2181-1) contains supplementary material, which is available to authorized users.

## Background

Next Generation Sequencing (NGS) and genotyping (NGG) technologies have revolutionized variant genotyping and now allow cost-effective and genome-wide characterization of genetic diversity in a growing number of species including non-model species [1].

In livestock species, based on low to high density SNP chips, a growing amount of genomic information on several dozens of local breeds have been generated as exemplified by cattle population studies [2–7] and studies in other species [8–10]. However, efficient storage of the huge resulting datasets for management, sharing and routine exploration purposes remains challenging.

We thus developed WIDDE, a Web accessible NoSQL Database, dedicated to the storage and management of dense genotyping datasets (e.g. up to hundreds of thousands of markers genotyped on thousands of individuals), coupled with various user friendly tools for (i) data selection, (ii) data exploration, (iii) export into various popular formats and (iv) population assignment. Via a web interface managing access to public (freely accessible) and private (accessible via login and password) data, users can therefore select (on a population and/or marker location basis) data subsets, perform basic data quality checking and standard population genetic analyses via a test for Hardy-Weinberg equilibrium and principal component analysis (PCA), and export the resulting datasets into various popular formats. Users can also jointly analyze their own genotyping data with WIDDE data subsets, in order to explore genetic proximity between populations by allele sharing distance (ASD) calculation, PCA and supervised clustering, to perform an estimation of ancestry composition of the samples and population assignment.

WIDDE functionalities are illustrated on a large and evolving cattle dataset which is representative of the world-wide bovine genetic diversity.

## Construction and content

### Database architecture and implementation details

**Fig. 1:**
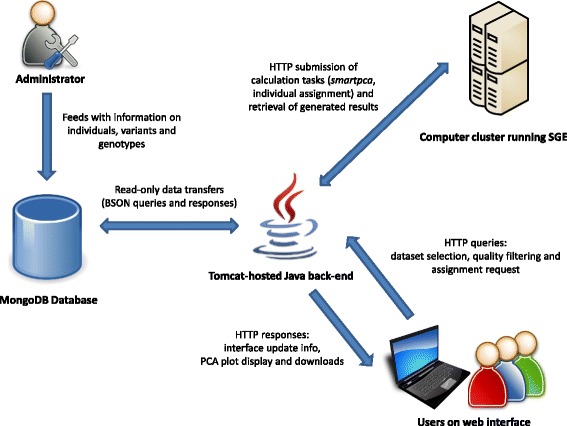
WIDDE architecture diagram. This high-level diagram illustrates the WIDDE architecture. It provides information about entities involved when using the information system, the data flows that occur between them, and the third-party software used in the process

The WIDDE architecture diagram is shown on Fig. 1. From a technical point of view, we used a NoSQL database engine to store and efficiently query millions of genotypes. MongoDB (http://www.mongodb.org/) was chosen as an open-source solution supporting complex queries and easy scalability. MongoDB achieves relationship management by providing a concept of collections able to store documents with a flexible structure and that can be embedded in one another. Thereby, it uses BSON (Binary JSON; http://bsonspec.org/) as data storage format. Defining a data structure for use with NoSQL relies on preliminary analysis of the queries that the targeted application will need to execute. Therefore, WIDDE data structure was centered on variants (Additional file 1: Figure S1) due to the higher expected number of variants to be stored compared to the number of individuals. The genotyping data documents are stored in a collection where keys consist in triplets (variant, project, run). Such documents, which are the most basic unit of data stored in the database, embed marker genotypes for all samples involved in the given run.

The server application was written in Java making use of the industry-standard Spring framework (http://spring.io/). Opal Toolkit (http://nbcr.ucsd.edu/data/docs/opal/) allows submitting jobs to a computer cluster running Sun Grid Engine (SGE), to perform either PCA or individual assignment. The client interface was developed in JSP with the jQuery JavaScript library (http://jquery.com/), and relies on the D3.js library for PCA result display (http://d3js.org/).

### Methods

PCA of individuals based on SNP genotyping data is performed with the *smartpca* software package [11].

Assignment of new individuals provided by users to WIDDE populations is performed using both distance method [12] and supervised clustering [13]. Allele sharing distance (ASD), defined as *1-x*_*ij*_ where *x*_*ij*_ represents the proportion of allele alike in state averaged over all genotyped SNPs, are calculated between individuals submitted by users and all public individuals included in WIDDE [6]. For each submitted individual, the average ASD with all individuals of each population is also calculated and the top 5 or 10 genetically closest populations are summarized.

Supervised clustering is used to estimate ancestry proportions of samples relative to each reference population represented in the database (world dataset). We relied on a simplified version of the EM algorithm described in [13] and [14] to estimate (genome-wide) ancestry proportions of each individual relative to the reference populations. To that end, we first estimate SNP allele frequencies within each reference population using a Laplace approximation: ${f}_i=\kern0.5em \frac{y_i+1}{n_i+2}$, where *y*_*i*_ is the allele count and *n*_*i*_ the total allele count for population i. Then, we used the likelihood model proposed by the FRAPPE’s EM algorithm [14] and Admixture to estimate the fraction *q*_*jk*_ of individual j’s genome assigned to the *k* populations (see equations 2 and 4 in [13]) using different values for the EM algorithm’s ε stopping criterion (0.01, 0.1 and 1). As the convergence of EM algorithm is slow, a fairly loose ε criterion is used to allow a fast termination of the algorithm. A smaller value of ε improves the accuracy of parameter estimates (providing the algorithm is not converging to a local optimum) at a cost of additional computational burden.

### Data source for WIDDE-cattle

The first WIDDE version includes bovine genotyping data, obtained with medium to high density Illumina SNP chips (54K and 770K), from different breeds arising from biodiversity studies [6, 7, 15–18]. Genotyping data from [6, 7, 15, 18] were already stored in the Dryad Digital Repository [19–21]. Genotyping data produced by Gautier & Naves were available as online supporting information [16] and data from [17] that we produced, were added to the database. HD genotyping data were obtained from Illumina.

Only cattle populations with at least 5 individuals were stored in WIDDE and those with at least 15 individuals were chosen as reference populations for population assignment. SNPchiMp was used to obtain consistent marker-lists and identify marker synonyms [22] and all markers were mapped on the current reference genome assembly bosTau6 UMD3.1. We also detected identical SNPs with different Illumina identifiers (98 duplicates and 6 triplicates) based on chromosome position. At these positions, we checked that the genotypes were identical for each individual and stored only one genotype per chromosome position in WIDDE. Each SNP stored in WIDDE is thus unique and ID synonym information was stored.

At the time of writing, final data imports into WIDDE of any new population can be proposed by contacting administrators.

## Utility and Discussion

### Application features

The WIDDE application has four main functionalities: (i) storing high density genotyping data for hundreds to thousands of individuals each characterized by their population of origin and genotyping projects, (ii) selecting, filtering and exporting genotyping data subsets in several formats (i.e. plink, eigenstrat, hapmap) for downstream analyses, (iii) exploring directly intra-species genetic diversity via PCA and (iv) exploring user-provided genotyped individuals with WIDDE individuals by assigning them to WIDDE reference populations. This latter step includes a visual assignment through PCA, a distance-based assignment without calibration and an estimation of samples’ ancestry composition relative to reference populations by supervised clustering [12–14]. WIDDE supports storing information from any type of markers derived from NGS (e.g. vcf file), NGG (SNP data) or older technologies (e.g. microsatellites data). Moreover, the WIDDE data structure contains various information about populations, genotyping projects, marker ID synonyms and chromosomal positions on current given reference genome assembly. WIDDE handles public and private (accessible via login/password) genotyping data.

### Web-interface

**Fig. 2:**
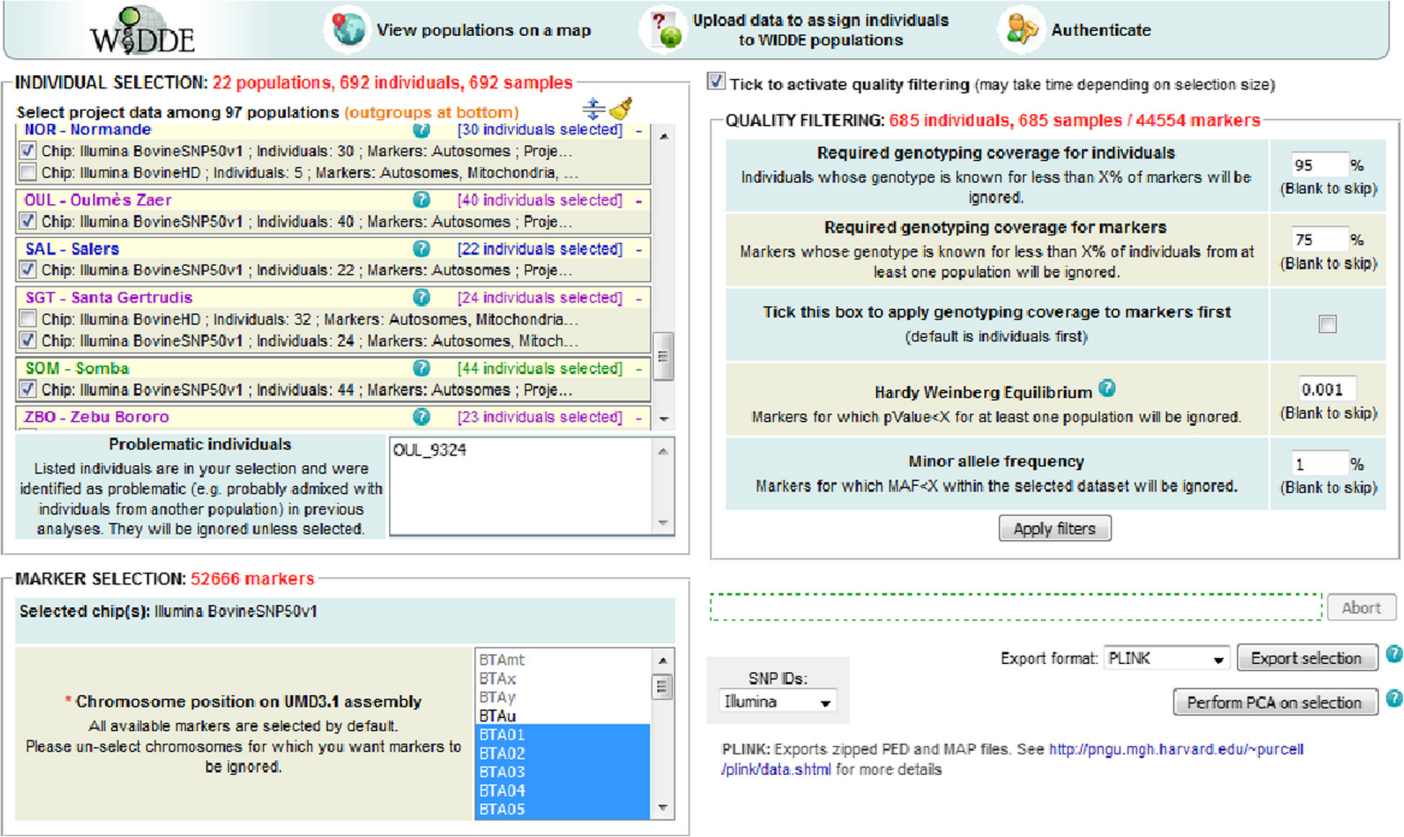
Web interface to select individuals and markers, apply quality filter, export data in various formats and launch principal component analysis

The WIDDE website consists in five sections accessible from the homepage. The “Home” section provides a concise description of the database and gives general information about the tool. The “Tutorial” section contains a didactic step-by-step tutorial illustrated with several screenshots. The “Data sources” section lists the different references and sources of data included in the database and the “Contact us” section contains the name, affiliation and email addresses of the main people involved in database conception and maintenance. The “Cattle data” section gives access to the actual application dedicated to bovine species. At the top of the application’s screen, a logo and three icons allow respectively to (i) return to the homepage, (ii) visualize populations’ origin on a map, (iii) upload data to launch population assignment and user’s genotyping data exploration, and (iv) authenticate to have access to private genotyping data. At the middle of the screen, a user friendly Web interface contains three panels for individual selection, marker selection and quality filtering, successively appearing when previous selection is valid (Fig. 2). Indeed, the dataset is defined in two steps. Individuals are selected from the first box, according to their population, genotyping project of origin and possible misidentification (e.g. problematic individuals identified by previous genetic analyses, due to population misidentification on phenotype). While choosing from the population list, the total numbers of currently selected individuals and samples are automatically displayed. A batch selection of individuals by population groups (European taurine, African taurine, zebu, hybrid and outgroup species) and DNA chip model (Illumina BovineSNP50v1, Illumina BovineSNP50v2 and Illumina BovineHD) is also possible. Selected chips are then displayed in the second box and markers can be selected according to their DNA location (mitochondrial, autosomal and/or sex chromosomes). The number of markers in the current selection is also kept up to date in real-time. An optional quality filtering step is available in a third box where two thresholds fix the minimum genotyping call rate for individuals and markers (95 % and 75 % by default). The order in which these two first filters are applied can be reversed by ticking another checkbox. By carrying out an exact test for Hardy-Weinberg Equilibrium [23], a third filter can be applied to discard outlying markers (*P* < 0.001 by default). Last, a filter on Minor Allele Frequency computed over all populations of the selected dataset can discard poorly informative markers (MAF < 0.01 by default).

Before or after quality filtering, genotyping data can be exported in popular formats (e.g. *plink* [24]*, eigenstrat* [25]).

**Fig. 3:**
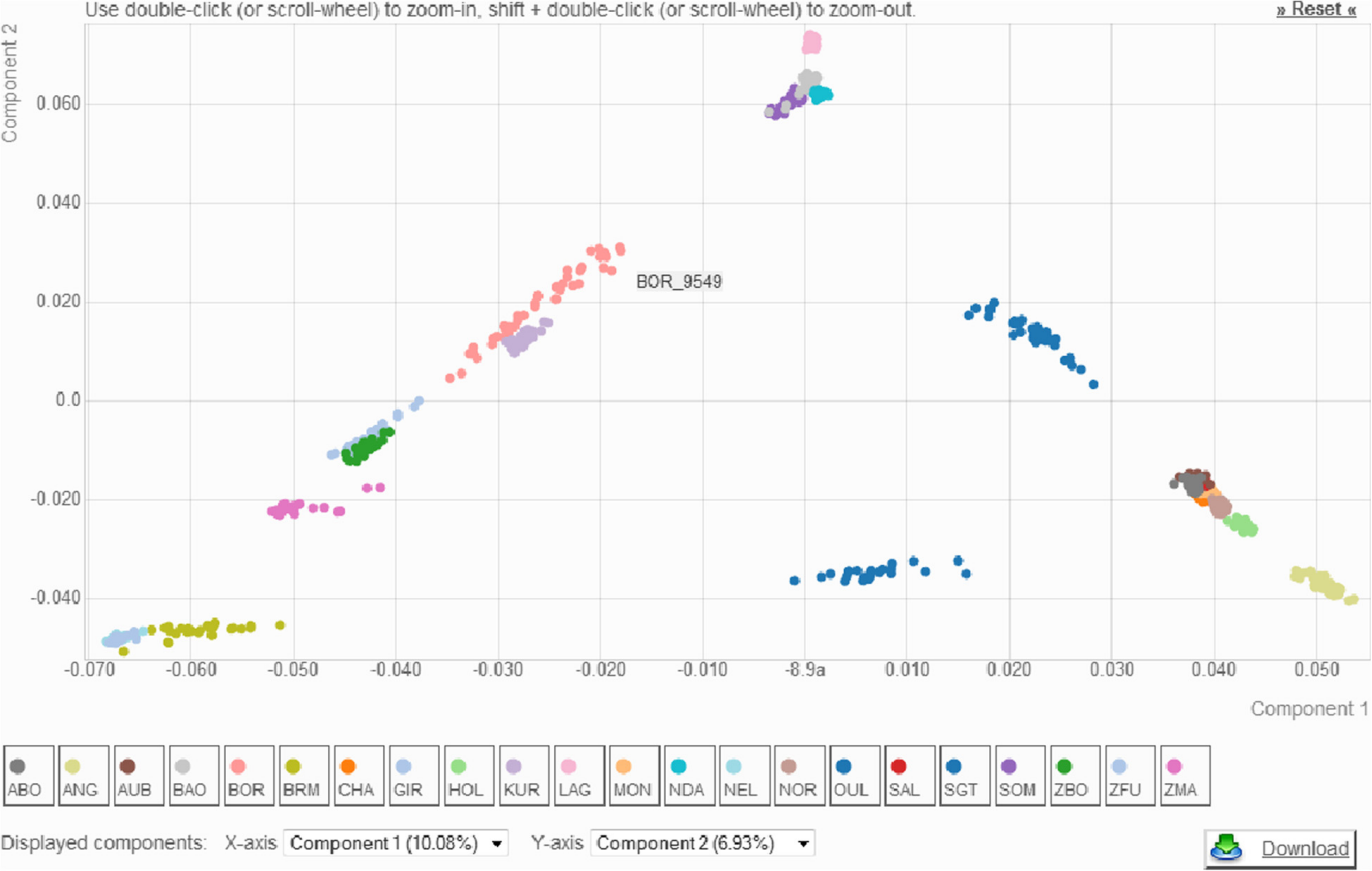
Plot of the individuals according to their coordinates on the first two principal components of the principal component analysis including 44,554 SNPs genotyped on 685 individuals from 22 cattle populations representative of the cattle genetic diversity. Eight EUT (Abondance/ABO, Angus/ANG, Aubrac/AUB, Charolais/CHA, Holstein/HOL, Montbéliard/MON, Normande/NOR and Salers/SAL), four AFT (Baoulé/BAO, Lagune/LAG, N’Dama/NDA and Somba/SOM), six ZEB (Brahman/BRM, Nelore/NEL, Gir/GIR, Zebu Bororo/ZBO, Zebu Fulani/ZFU and Zebu from Madagascar/ZMA) and four admixed populations (Borgou/BOR, Kouri/KUR, Oumes Zaër/OUL and Santa Gertrudis/SGT) genotyped on the Illumina Bovine SNP50v1 were selected. Data has been filtered using default parameters

Users may also explore genetic diversity via an online PCA performed with the *smartpca* software [11]. The dataset is first converted to eigenstrat format and *smarpca* is then launched on a computer cluster. Individuals are then plotted by default on the first factorial plan in a new window allowing selection of other components (Fig. 3). As this step may take time (few to few dozens of minutes depending on the size of the dataset and the cluster queue status), users have the possibility to enter their email address to be informed of the job completion. After job completion, genotyping data in *eigenstrat* format, a summary of individual and marker selection (*selection.txt*) as well as *smartpca* output files (*output.pca.evec*, *output.eval* and *sdtout.txt*) may be downloaded.

By clicking on the assignment icon, users also have the option through an upload interface to analyze their own genotyping data (in *plink* format with nucleotide letters) with WIDDE public genotyping data. This process, which can be time consuming, is also detached from the web interface and runs on the mentioned computer cluster via Opal and SGE. Based on SNPs in common, user can choose (i) to perform a PCA of genotyping data combined with public genotyping data stored in WIDDE, and (ii) to assign these new individuals to populations of the public reference dataset based on ASD calculation and on estimation of ancestry proportions by supervised clustering [12–14]. ASD between submitted individuals and each individual from the reference dataset are calculated and the top five or ten populations of the WIDDE reference dataset with the weakest ASD average are listed (along with ASD minimum and maximum within populations) for each new individual. The supervised clustering step determines for each new individual the proportion of ancestry attributed to each population of the WIDDE reference dataset. Users, who may enter their email address to be informed of job completion, can download: (i) a summary and the complete results of ASD calculation (*asd_summary.tsv* and *asd_results.tsv*), (ii) a summary and the complete results of the supervised clustering (*ancestry_summary.tsv* and *ancestry_results.tsv*) and (iii) the merged dataset used in the analysis in *eigenstrat* format.

### Illustration with cattle data

As an illustration of WIDDE functionalities, we hereby detailed the cattle module currently containing 783,640 SNPs and 3951 (2827 publicly available) individuals belonging to 129 (89 public) different cattle populations and 8 (7 public) populations of outgroup species (two *Bos javanicus* populations, *Bison bison*, *Syncerus caffer*, *Bos gaurus*, *Bubalus depressicornis*, *Bos grunniens*), that were thoroughly selected and curated. These various local cattle populations are representative of the bovine genetic diversity and belong to the three main cattle groups, i.e. European (EUT) and African (AFT) taurine (*Bos taurus*) and zebus (ZEB; *Bos indicus*). Figure 3 describes the PCA results of a data subset including 685 individuals from 22 cattle populations representative of EUT, AFT and ZEB, genotyped on 44,554 SNPs after quality filtering using default settings (Fig. 2). The first factorial plan allows recovering the already described triangle-like 2-dimensional global organization of cattle genetic diversity [6]. Briefly, each main cattle group is positioned at the three apexes of the triangle and admixed populations lie at intermediate positions.

**Fig. 4:**
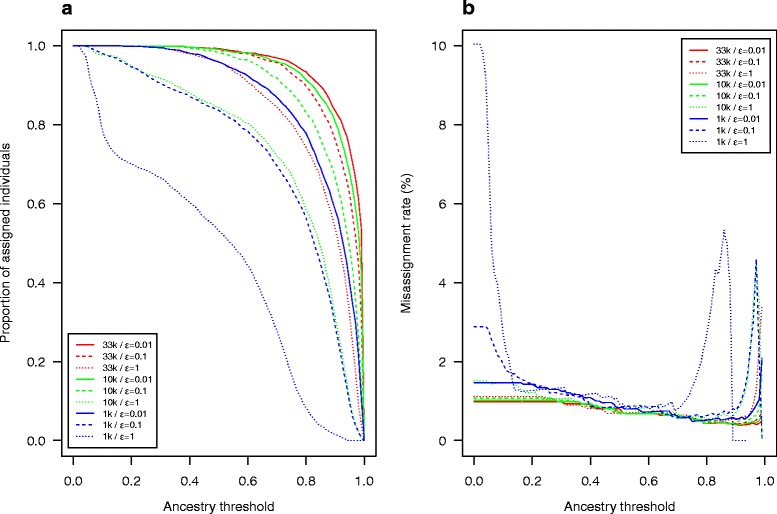
Proportion of assigned individuals and misassignment rate in assignment tests based on supervised clustering. The 2250 individuals from 45 public populations of the world reference dataset were assigned against the world reference dataset, using 32,966 (33K) SNP, 10K SNP and 1K SNP, with different values for the EM algorithm’s ε stopping criterion (0.01, 0.1 and 1). The proportion of assigned individuals **a** and the misassignment rate **b** were plotted against ancestry thresholds (0–1)

A world reference dataset including all WIDDE public populations representative of the world-wide genetic diversity of the bovine species with at least 15 individuals was defined to assign user-uploaded individuals to WIDDE public populations. To illustrate this step, we estimated ancestry proportions of 2250 individuals from 45 public populations of the world reference dataset against the world reference dataset itself using supervised clustering. We started from 33K SNP (i.e. the highest number of variants taken into account in the analysis), 10K SNP and 1K SNP randomly chosen within the 33K list, and considered different EM stopping criteria (ε = 0.01, ε = 0.1 and ε = 1). Based on these supervised clustering results, the proportion of assigned individuals and the misassignment rate were then calculated for different ancestry thresholds ranging from 0 to 1 (Fig. 4). More precisely, for a given threshold t, each individual was assigned to a population j if the estimated ancestry proportion *q*_*ij*_ > t (for a small value of t, if several populations satisfied this criterion, the individual was assigned to the population displaying the highest ancestry proportion). As a result, the assignment rate (for a given ancestry threshold t) was defined as the proportion of individuals assigned to a population and the misassignment rate corresponded to the proportion (with respect to all the assigned individuals) that were assigned to a population different from their population of origin.

As expected, the proportion of assigned individuals increased with the number of selected markers and with the stringency of the stopping criterion (Fig. 4a). For instance, at an ancestry threshold of 0.8, the proportions of assigned individuals were above 80 % (respectively 60 %) with 33K (respectively 10K) SNPs whatever the stopping criterion value. Conversely, the misassignment rates always remained under 2 % with both 33K and 10K SNPs (Fig. 4b). Note however that for a small number of SNPs (e.g. 1K) the misassignment rates sometimes reached values above 5 %. As a rule of thumb, one may thus recommend using for assignment purposes at least 10K SNPs and an ancestry threshold above 0.75.

We also applied WIDDE diversity exploration and population assignment tools to a test dataset with individuals not included in the database and belonging to two European taurine breeds i.e. Montbeliard (2 individuals) and Tarentaise (5 individuals) [7]. Tarentaise is simply another name for the Tarine although considered as a separate breed in [7]. We first checked that these populations were positioned near the EUT group as expected on the first factorial plan of PCA (Additional file 2: Figure S2). In order to have an idea of the WIDDE populations presenting the strongest genetic proximity with the uploaded individuals, we then launched the assignment module using the reference dataset (ε = 0.01). Additional file 3: Tables S1 and S2 resume for each new individual the top five nearest WIDDE populations based on average ASD calculation and the proportion of ancestry attributed to each population of the reference dataset, respectively. We thus checked that the Montbéliard and Tarentaise individuals were properly assigned as the corresponding populations were already present in WIDDE. The two supposed Montbéliard individuals presented the closest genetic distance with MON with a proportion of ancestry above 95 %, as expected (Additional file 3: Tables S1 and S2). The supposed Tarentaise individuals proved genetically close to TAR (Tarine) population but with a weaker proportion of ancestry attributed to this breed (between 48.8 and 68.7 %), illustrating a possible admixture with other European taurine breeds.

Our results demonstrate the utility of WIDDE in assigning any individual to the genetically closest population and the ability to estimate the ancestry proportion of any individual to the WIDDE reference populations*.* The assignment method based on supervised clustering is especially accurate when the true population of origin is included in the reference dataset [12]. When the new individual’s true breed population is not present in WIDDE, the tool still provides an estimate of the mostly closely related population. Future work will require the implementation of an exclusion method to measure the confidence that an individual truly belongs to a given population. As these exclusion methods are at the moment difficult to implement with an acceptable computation time compatible with a high number of markers, they were not integrated into WIDDE but output for *Geneclass 2.0* software might be easy to generate and will be available shortly [26].

## Conclusion

In summary, the NoSQL next generation database WIDDE represents a biodiversity database able to manage and explore a large amount of genotyping data, and to assign new individuals to populations stored internally. Thus, WIDDE is a generic tool applicable to a wide range of species and marker types. It is a versatile tool and further version of the database will include additional output formats and new tools dedicated to genetic diversity exploration. The first module, WIDDE-cattle, described here, represents the first database dedicated to cattle biodiversity and SNP genotyping data, which allows users to explore not only the WIDDE dataset but also their own genotyping data. It will be very useful for researchers interested in cattle genetic diversity and will be extended to other livestock species shortly.

## Availability and requirements

WIDDE is deployed on our institutional website at http://widde.toulouse.inra.fr for research and academic use.

## Additional files

Additional file 1: Figure S1. Sample BSON documents illustrating variant and genotyping data storage. The two typical kinds of database records, i.e. Variant document and VariantRunData document, contain variant-level information and run-level genotyping data, respectively. Because storage is made in such a way that dictionary keys are repeated for each record, we defined them as short as two characters in order to keep disk space usage reasonable. Flexibility is illustrated by the use of lists and dictionaries, which can grow without the need to redefine a fixed model. (PDF 160 kb) Additional file 2: Figure S2. Plot of the individuals according to their coordinates on the first two principal components of the principal component analysis including 31,477 SNPs genotyped on 2270 individuals from 49 cattle populations. This dataset contains 7 new individuals from two populations i.e. Montbéliard (A_221) and Tarentaise (A_125). The individuals which belong to the three main cattle population groups (European taurine, African taurine and Zebu) are indicated on the plot. (PDF 157 kb) Additional file 3: Table S1. Summary of average allele sharing distance results obtained for the new individuals belonging to Tarentaise and Montbéliard populations. The top five averages allele sharing distance calculated between 7 new individuals belonging to Tarentaise (125) and Montbéliard (221) populations and the 2250 individuals from the 45 public populations of the WIDDE reference dataset are presented. For each new individual, the minimum and maximum allele sharing distances per reference population are also mentioned. **Table S2.** Summary of the supervised clustering results obtained for the 7 new individuals belonging to Tarentaise (125) and Montbéliard (221) populations. For each individual, the different proportions of ancestry above 1 % attributed to a reference population are indicated in descending order. (XLSX 14 kb)

## References

[1] Davey JW, Hohenlohe PA, Etter PD, Boone JQ, Catchen JM, Blaxter ML (2011). Genome-wide genetic marker discovery and genotyping using next-generation sequencing. Nat Rev Genet.

[2] Decker JE, Pires JC, Conant GC, McKay SD, Heaton MP, Chen K (2009). Resolving the evolution of extant and extinct ruminants with high-throughput phylogenomics. Proc Natl Acad Sci U S A.

[3] Elsik CG, Tellam RL, Worley KC, Gibbs RA, Muzny DM, Weinstock GM (2009). The genome sequence of taurine cattle: a window to ruminant biology and evolution. Science.

[4] Flori L, Fritz S, Jaffrezic F, Boussaha M, Gut I, Heath S (2009). The genome response to artificial selection: a case study in dairy cattle. PLoS One.

[5] Gautier M, Flori L, Riebler A, Jaffrezic F, Laloe D, Gut I (2009). A whole genome Bayesian scan for adaptive genetic divergence in West African cattle. BMC Genomics..

[6] Gautier M, Laloe D, Moazami-Goudarzi K. Insights into the genetic history of French cattle from dense SNP data on 47 worldwide breeds. PLoS One. 2010;5(9):e13038. 10.1371/journal.pone.0013038 PMC2948016 20927341

[7] Decker JE, McKay SD, Rolf MM, Kim J, Molina Alcala A, Sonstegard TS (2014). Worldwide patterns of ancestry, divergence, and admixture in domesticated cattle. PLoS Genet.

[8] Kijas JW, Lenstra JA, Hayes B, Boitard S, Porto Neto LR, San Cristobal M (2012). Genome-wide analysis of the world’s sheep breeds reveals high levels of historic mixture and strong recent selection. PLoS Biol.

[9] Wilkinson S, Lu ZH, Megens HJ, Archibald AL, Haley C, Jackson IJ (2013). Signatures of diversifying selection in European pig breeds. PLoS Genet.

[10] Tosser-Klopp G, Bardou P, Bouchez O, Cabau C, Crooijmans R, Dong Y (2014). Design and characterization of a 52K SNP chip for goats. PLoS One.

[11] Patterson N, Price AL, Reich D (2006). Population structure and eigenanalysis. PLoS Genet.

[12] Cornuet JM, Piry S, Luikart G, Estoup A, Solignac M (1999). New methods employing multilocus genotypes to select or exclude populations as origins of individuals. Genetics.

[13] Alexander DH, Novembre J, Lange K (2009). Fast model-based estimation of ancestry in unrelated individuals. Genome Res.

[14] Tang H, Peng J, Wang P, Risch NJ (2005). Estimation of individual admixture: analytical and study design considerations. Genet Epidemiol.

[15] Matukumalli LK, Lawley CT, Schnabel RD, Taylor JF, Allan MF, Heaton MP (2009). Development and characterization of a high density SNP genotyping assay for cattle. PLoS One.

[16] Gautier M, Naves M (2011). Footprints of selection in the ancestral admixture of a New World Creole cattle breed. Mol Ecol.

[17] Flori L, Gonzatti MI, Thevenon S, Chantal I, Pinto J, Berthier D (2012). A quasi-exclusive European ancestry in the Senepol tropical cattle breed highlights the importance of the slick locus in tropical adaptation. PLoS One.

[18] Flori L, Thevenon S, Dayo GK, Senou M, Sylla S, Berthier D (2014). Adaptive admixture in the West African bovine hybrid zone: insight from the Borgou population. Mol Ecol.

[19] Gautier M, Laloë D, Moazami-Goudarzi K. Data from: Insights into the genetic history of French cattle from dense SNP data on 47 worldwide breeds. Dryad Digital Repository 2010. http://dx.doi.org/10.5061/dryad.2f185. Accessed 25 sept 2012. 10.1371/journal.pone.0013038 PMC2948016 20927341

[20] Flori L, Thévenon S, Dayo GK, Senou M, Sylla S, Berthier D, et al. Data from: Adaptive admixture in the West African bovine hybrid zone: insight from the Borgou population. Dryad Digital Repository. 2014. http://dx.doi.org/10.5061/dryad.281f2. Accessed 3 June 2014. 10.1111/mec.12816 24888437

[21] Decker JE, McKay SD, Rolf MM, Kim J, Alcala AM, Sonstegard TS, et al. Data from: Worldwide patterns of ancestry, divergence, and admixture in domesticated cattle. Dryad Data Repository. 2014. http://dx.doi.org/10.5061/dryad.th092. Accessed 17 April 2014. 10.1371/journal.pgen.1004254 PMC3967955 24675901

[22] Nicolazzi EL, Picciolini M, Strozzi F, Schnabel RD, Lawley C, Pirani A (2014). SNPchiMp: a database to disentangle the SNPchip jungle in bovine livestock. BMC Genomics..

[23] Wigginton JE, Cutler DJ, Abecasis GR (2005). A note on exact tests of Hardy-Weinberg equilibrium. Am J Hum Genet.

[24] Purcell S, Neale B, Todd-Brown K, Thomas L, Ferreira MA, Bender D (2007). PLINK: a tool set for whole-genome association and population-based linkage analyses. Am J Hum Genet.

[25] Price AL, Patterson NJ, Plenge RM, Weinblatt ME, Shadick NA, Reich D (2006). Principal components analysis corrects for stratification in genome-wide association studies. Nat Genet.

[26] Piry S, Alapetite A, Cornuet JM, Paetkau D, Baudouin L, Estoup A (2004). GENECLASS2: a software for genetic assignment and first-generation migrant detection. J Hered.

