# Supplementary material for: WIDDE: a Web-Interfaced next generation database for genetic diversity exploration, with a first application in cattle
Source: BMC Genomics. 2015 Nov 14;16:940. doi: 10.1186/s12864-015-2181-1 (PMC4647285; doi:10.1186/s12864-015-2181-1)
Supplement: Additional file 1: Figure S1. — Sample BSON documents illustrating variant and genotyping data storage. The two typical kinds of database records, i.e. Variant document and VariantRunData document, contain variant-level information and run-level genotyping data, respectively. Because storage is made in such a way that dictionary keys are repeated for each record, we defined them as short as two characters in order to keep disk space usage reasonable. Flexibility is illustrated by the use of lists and dictionaries, which can grow without the need to redefine a fixed model. (PDF 160 kb) [file 12864_2015_2181_MOESM1_ESM.pdf]

Example of a Variant document (BSON format), containing information about the marker itself

```
{
  "_id" : "BTA000628179",_______________Variant ID, unique per variant type and position on the genome
  "_class" : "VD",______________________Required by Spring-Data, used for Object/Document Mapping
  "v" : NumberLong(3),__________________Variant records are versioned to achieve optimistic locking during imports
  "ty" : "SNP",_________________________Variant type code
  "sy" : {______________________________Block containing synonyms for this variant, grouped by category
    "il" : ["ARS-BFGL-NGS-116023", "BFGL-NGS-116023"],______________List of Illumina synonyms for this variant
    "in" : ["F0407890"],________________List of synonyms stored for internal use
    "nc" : ["rs109318750"]______________List of NCBI RefSNP (rs) synonyms
    },
    "ka" : ["G", "A"]___________________List of known alleles for this variant
}
```

Example of a VariantRunData document (BSON format), embedding variant genotypes for all samples involved in a run

```
{
    "_id" : {___________________________VariantRunData ID (composite)
        "pi" : NumberInt(10), __________Project ID
        "rn" : "run1", _________________Run name
        "vi" : "BTA000628179"___________Variant ID
    },
    "_class" : "R", ____________________Required by Spring-Data, used for Object/Document Mapping
    "sp" : { ___________________________List of samples involved in this run
        "1" : {_________________________Block containing data related to the genotyping of sample 1 in this run
            "gt" : "1/1"________________Genotype found for sample 2 in this run: A/A
        },
        "2" : {_________________________Block containing data related to the genotyping of sample 2 in this run
            "gt" : "0/0"________________Genotype found for sample 2 in this run: G/G
        },
        "3" : {_________________________Block containing data related to the genotyping of sample 3 in this run
            "gt" : "0/1"________________Genotype found for sample 2 in this run: G/A
        }

    }
}
```
