# Supplementary material for: WIDDE: a Web-Interfaced next generation database for genetic diversity exploration, with a first application in cattle
Source: BMC Genomics. 2015 Nov 14;16:940. doi: 10.1186/s12864-015-2181-1 (PMC4647285; doi:10.1186/s12864-015-2181-1)
Supplement: Additional file 2: Figure S2. — Plot of the individuals according to their coordinates on the first two principal components of the principal component analysis including 31,477 SNPs genotyped on 2270 individuals from 49 cattle populations. This dataset contains 7 new individuals from two populations i.e. Montbéliard (A_221) and Tarentaise (A_125). The individuals which belong to the three main cattle population groups (European taurine, African taurine and Zebu) are indicated on the plot. (PDF 157 kb) [file 12864_2015_2181_MOESM2_ESM.pdf]

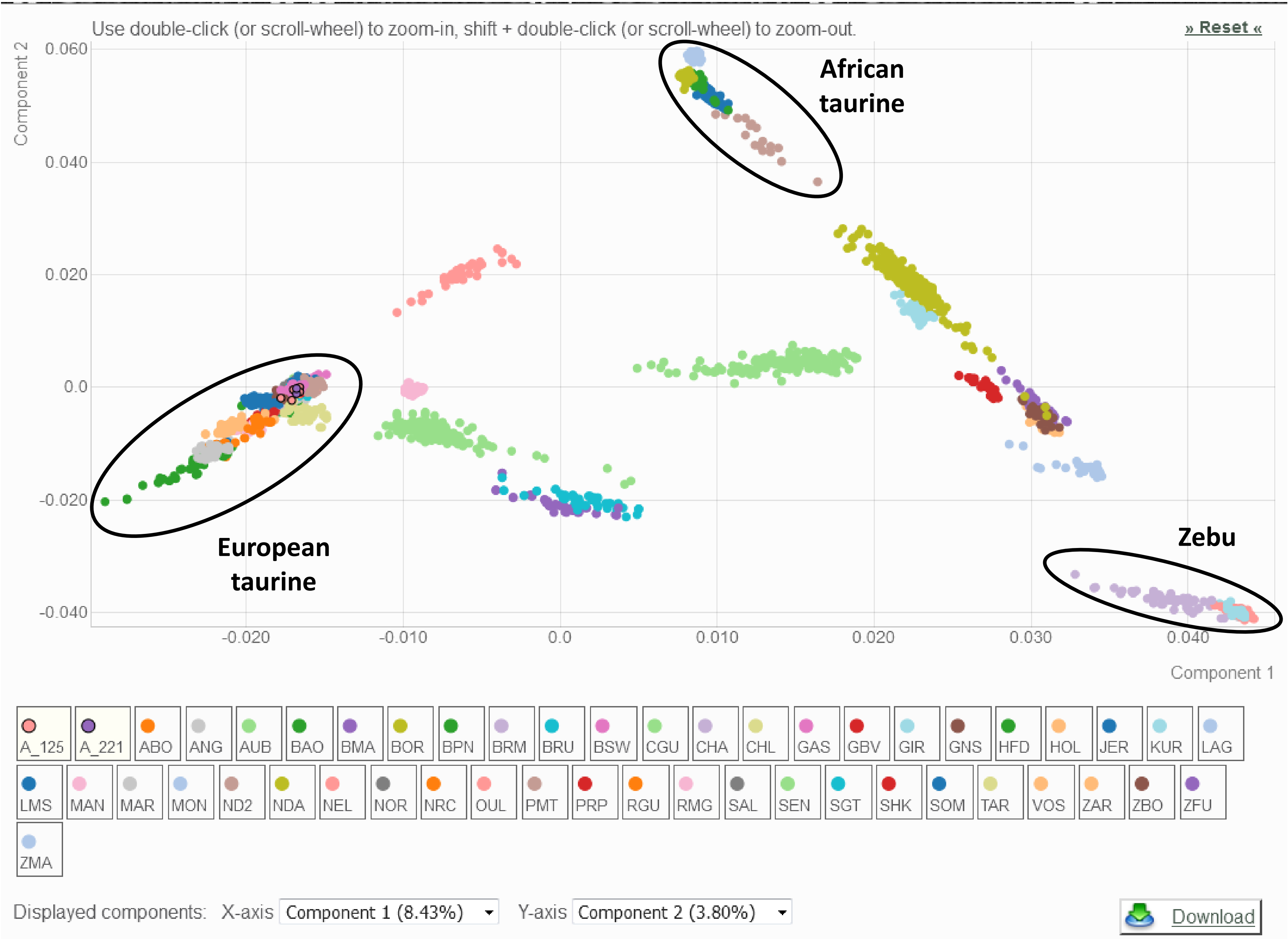

Use double-click (or scroll-wheel) to zoom-in, shift + double-click (or scroll-wheel) to zoom-out.
» Reset «
Component 2
0.060
0.040
0.020
0.0
-0.020
-0.040
-0.020
-0.010
0.0
0.010
0.020
0.030
0.040
Component 1
African taurine
European taurine
Zebu
A_125
A_221
ABO
ANG
AUB
BAO
BMA
BOR
BPN
BRM
BRU
BSW
CGU
CHA
CHL
GAS
GBV
GIR
GNS
HFD
HOL
JER
KUR
LAG
LMS
MAN
MAR
MON
ND2
NDA
NEL
NOR
NRC
OUL
PMT
PRP
RGU
RMG
SAL
SEN
SGT
SHK
SOM
TAR
VOS
ZAR
ZBO
ZFU
ZMA
Displayed components: X-axis Component 1 (8.43%) Y-axis Component 2 (3.80%)
Download
